# Predicting procedure duration of colorectal endoscopic submucosal dissection at Western endoscopy centers

**DOI:** 10.1055/a-2122-0419

**Published:** 2023-08-07

**Authors:** Hao Dang, Nik Dekkers, Ewout W. Steyerberg, Francisco Baldaque-Silva, Masami Omae, Krijn J.C. Haasnoot, Laurelle van Tilburg, Kate Nobbenhuis, Jolein van der Kraan, Alexandra M.J. Langers, Jeanin E. van Hooft, Wilmar de Graaf, Arjun D. Koch, Paul Didden, Leon M.G. Moons, James C.H. Hardwick, Jurjen J. Boonstra

**Affiliations:** 1 4501 Gastroenterology and Hepatology, Leiden University Medical Center, Leiden, Netherlands; 2 4501 Biomedical Data Sciences, Leiden University Medical Center, Leiden, Netherlands; 3 59562 Endoscopy Unit, Center for Upper Digestive Diseases, Karolinska Hospital, Stockholm, Sweden; 4 37824 Advanced Endoscopy Center Carlos Moreira da Silva, Pedro Hispano Hospital, Matosinhos, Portugal; 5 8124 Gastroenterology & Hepatology, University Medical Centre Utrecht, Utrecht, Netherlands; 6 6993 Gastroenterology and Hepatology, Erasmus MC, Rotterdam, Netherlands; 7 8124 Gastroenterology and Hepatology, University Medical Centre Utrecht, Utrecht, Netherlands

**Keywords:** Polyps / adenomas / ..., Endoscopic resection (polypectomy, ESD, EMRc, ...), Endoscopy Lower GI Tract, Colorectal cancer, Quality and logistical aspects, Preparation

## Abstract

**Background and study aims** Overcoming logistical obstacles for the implementation of colorectal endoscopic submucosal dissection (ESD) requires accurate prediction of procedure times. We aimed to evaluate existing and new prediction models for ESD duration.

**Patients and methods** Records of all consecutive patients who underwent single, non-hybrid colorectal ESDs before 2020 at three Dutch centers were reviewed. The performance of an Eastern prediction model [GIE 2021;94(1):133–144] was assessed in the Dutch cohort. A prediction model for procedure duration was built using multivariable linear regression. The model’s performance was validated using internal validation by bootstrap resampling, internal-external cross-validation and external validation in an independent Swedish ESD cohort.

**Results** A total of 435 colorectal ESDs were analyzed (92% en bloc resections, mean duration 139 minutes, mean tumor size 39 mm). The performance of current unstandardized time scheduling practice was suboptimal (explained variance: R ^2^ =27%). We successfully validated the Eastern prediction model for colorectal ESD duration <60 minutes (c-statistic 0.70, 95% CI 0.62–0.77), but this model was limited due to dichotomization of the outcome and a relatively low frequency (14%) of ESDs completed <60 minutes in the Dutch centers. The model was more useful with a dichotomization cut-off of 120 minutes (c-statistic: 0.75; 88% and 17% of “easy” and “very difficult” ESDs completed <120 minutes, respectively). To predict ESD duration as continuous outcome, we developed and validated the six-variable cESD-TIME formula ( https://cesdtimeformula.shinyapps.io/calculator/ ; optimism-corrected R ^2^ =61%; R ^2^ =66% after recalibration of the slope).

**Conclusions** We provided two useful tools for predicting colorectal ESD duration at Western centers. Further improvements and validations are encouraged with potential local adaptation to optimize time planning.

## Introduction

Colorectal endoscopic submucosal dissection (ESD) is an endoscopic resection technique which is widely used in Eastern countries. However, it has been slow to be taken up by Western endoscopists 1 2 . This is mainly due to a lack of local experts and opportunities to acquire sufficient skill in ESD, which makes the procedure complex and time-consuming for them. Several studies demonstrated that for equivalent-sized lesions, the procedural time for ESD may exceed the time needed for piecemeal endoscopic mucosal resection (pEMR) by three to four times 3 4 . This introduces logistical problems in Western endoscopy centers because the shortage of resources does not allow for the routine use of such time-consuming procedures that are difficult to learn. Nevertheless, colorectal ESD is gradually gaining ground in the West because it enables accurate histological staging and decreases the risk of recurrence 5 , thereby also reducing the need for intensive follow-up programs and additional endoscopic or surgical treatment after the initial endoscopic resection.

To overcome the logistical obstacles for implementing colorectal ESD, it is essential to anticipate possible long procedure durations by accurate time scheduling. In this study, we evaluated the performance of current time planning practice and a previous Eastern prediction model 6 for colorectal ESD duration, and developed a new model with procedure time as continuous prediction outcome.

## Patients and methods

This study is reported in accordance with the TRIPOD statement ( **Supplementary Checklist** ) and was approved by the Medical Ethical Committee of Leiden University Medical Center (reference number G18.097). The variable definitions, ESD endoscopists/procedures and statistical analyses are detailed in the **Supplementary Methods** .

### Patients

The analysis cohort consisted of consecutive patients who underwent ESD for colorectal neoplasms before January 1, 2020 at three Dutch tertiary endoscopy centers (Leiden University Medical Center, University Medical Center Utrecht, Erasmus MC Cancer Institute in Rotterdam). We included all cases starting from the first ESD ever performed, with the aim of making our findings also generalizable to beginning Western ESD endoscopists, the target group where the greatest benefit can be attained 7 . We excluded hybrid ESDs and procedures with two or more simultaneous ESDs. In addition, resections of subepithelial tumors (e.g. gastrointestinal stroma cell tumors, neuroendocrine tumors, lipomas), anal intraepithelial neoplasms and post-polypectomy scar resections by ESD were also excluded. Lastly, we excluded intended ESD procedures that were terminated prematurely without (complete) lesion removal. This was because in all these cases (n=43) the reason for premature ESD termination turned out to be deep tumor invasion (i.e. suspected invasion into the muscularis propria during the procedure), which made it impossible or futile to continue the resection. The vast majority of deeply invasive tumors could be anticipated pre-procedurally by optical diagnosis 8 . As we were interested in procedure durations resulting from the complexity of the ESD (and not from patient selection-related factors), we reasoned that including prematurely terminated ESDs would distort the analyses (e.g. relatively short procedure durations for very large but deeply invasive tumors). We chose not to exclude intended ESDs that were converted to piecemeal resection, as in general, endoscopists are reluctant to do so without first trying their utmost to achieve en bloc resection by ESD. The analysis cohort was used for descriptive statistics, evaluation of ESD duration-related outcomes, and development of a prediction model for colorectal ESD duration.

External validation of the developed prediction model was performed in an independent cohort of consecutive colorectal ESDs from a Swedish tertiary endoscopy center (Karolinska Institute, Stockholm). Parts of this cohort have been described previously 9 10 . Single, non-hybrid ESDs were selected using the above inclusion and exclusion criteria.

### Time planning analyses

The amount of time scheduled was decided for each case individually by or in consultation with the endoscopist performing the ESD. Procedure time was not scheduled in a standardized manner. Actual procedure duration was defined as the time between first introduction and final removal of the endoscope (the total “scope time”: i.e. including possible cleaning of the lesion and marking of its perimeter, dissection and retrieval of the resected specimen; excluding induction and recovery time of propofol sedation). When the actual ESD duration was >1 hour longer or shorter than the amount of time scheduled, endoscopy reports were extensively reviewed for possible explanations for the difference between the scheduled and actual time.

We also evaluated the performance of a previously published Eastern prediction model for colorectal ESD duration 6 (see **Supplementary Methods** for details). The predicted outcome of the model is ESD completion <60 minutes (yes/no). For sensitivity analyses, we also varied the dichotomization cut-off of the outcome with 1-minute increments over the entire range of ESD durations in our analysis cohort. For each cut-off, the model’s performance was evaluated.

### Model development

As the abovementioned Eastern model 6 used a dichotomized outcome, we built a new prediction model, the cESD-TIME formula, with colorectal ESD duration as continuous outcome. The initial formula consisted of the four variables in the Eastern model (tumor size, circumference, location and morphology). Besides, we also considered other pre-procedural variables that have been reported to be associated with the presence of submucosal fibrosis, a factor that could considerably increase ESD difficulty and duration 11 12 13 14 . These pre-procedural variables included the type of lesion (recurrent or naïve lesion to be resected) 15 16 , presence of a depressed area 17 18 , suspected invasive cancer 16 , prior biopsies taken 16 19 , and inflammatory bowel disease 20 21 . Lastly, we included the consecutive colorectal ESD number for each endoscopist in the model as a proxy for ESD experience, an important determinant of procedure duration 22 23 24 . The adopted ESD method was not considered in model development, because a randomized trial found no significant difference in dissection speed between the two ESD methods (conventional and pocket-creation method) that were used in the analysis cohort 25 .

As exploratory analysis, we also included endoscopic maneuverability in the cESD-TIME formula and evaluated how the model performance would change. Maneuverability is generally referred to as the degree of having difficulty with obtaining and maintaining an optimal dissection plane through the submucosa, e.g. due to paradoxical movement of the endoscope 7 26 27 . However, there are currently no standardized criteria available for determining or classifying endoscopic maneuverability. In this study, maneuverability was subjectively evaluated by the endoscopist during (and not before) the procedure, and was described as “good,” “average” or “poor.”

## Results

### Full cohort characteristics

In total, 435 single, non-hybrid colorectal ESDs performed by six endoscopists (#1: n=85, #2: n=39; #3: n=151, #4: n=8; #5: n=122, #6: n=30) were eligible for analysis ( **Supplementary Fig.1** ). Patient, lesion, and procedure characteristics and outcomes are shown in **Supplementary Table 1** , Table 1 , Table 2 , and Table 3 . The mean age was 67 years (SD 8.7), and the majority of patients undergoing ESD were male (62%). The mean lesion size was 39 mm (SD 25). Most lesions were located in the rectum (58%), followed by the left (35%) and right hemicolon (7.8%). A total of 94 ESDs (22%) were performed between 2011 and 2015, and 341 (78%) between 2016 and 2019. Throughout the entire study period, no traction device-assisted ESD techniques were used. The mean time scheduled for the ESD was 137 minutes (SD 53), and the actual procedure duration was 139 minutes (SD 95). En bloc resection was achieved in 402 of 435 cases (92%). ESD was converted to piecemeal resection in 33 cases (7.6%). The rate of ESD-related perforations requiring surgery was eight of 435 patients (1.8%). The R0 resection rate among en bloc resections was 69%. Histological evaluation of these specimens revealed invasive cancer in 145 cases (36%). Additional surgery after ESD was indicated in 87 cases (22%), mostly due to the presence of histological high-risk features. After a mean follow-up of 21 months, recurrence was found in four of 257 patients (1.6%) treated with en bloc ESD.

**Table:** **Table 1** Lesion characteristics of the analysis cohort (n=435 procedures).

	All centers n=435	Center #1 n=124	Center #2 n=159	Center #3 n=152	P value
Location					<0.001
Right hemicolon	34 (7.8)	1 (0.8)	19 (11.9)	14 (9.2)	
Cecum	6	0	6	0	
Hepatic flexure	5	0	3	2	
Splenic flexure	1	0	1	0	
Left hemicolon	150 (34.5)	36 (29.0)	52 (32.7)	62 (40.8)	
Junction of the sigmoid and descending colon*	8	1	1	6	
Rectosigmoid	48	22	12	16	
Rectum	251 (57.9)	87 (70.2)	88 (55.3)	76 (50.0)	
Lesion extending to the dentate line*	46	18	12	16	
Recurrence to be resected	22 (5.1)	3 (2.4)	8 (5.0)	11 (7.2)	0.19
Gross morphology*					<0.001
Pedunculated	25 (5.9)	1 (0.8)	24 (15.9)	0 (0.0)	
Sessile	241 (57.1)	78 (62.9)	95 (62.9)	68 (46.3)	
Flat	156 (37.0)	45 (36.3)	32 (21.2)	79 (53.7)	
Presence of a depressed area*	140 (33.3)	13 (10.7)	89 (59.7)	38 (25.3)	<0.001
Non-granular surface*	70 (34.1)	16 (53.3)	10 (22.7)	44 (33.6)	0.024
Lesion size, mean (SD), mm*	38.9 (24.5)	48.4 (23.6)	30.4 (23.4)	39.2 (23.5)	<0.001
Luminal circumference, mean (SD), %*	45.8 (20.1)	48.2 (20.3)	46.1 (22.7)	43.1 (18.6)	0.17
Suspected CRC in lesion*	244 (56.4)	65 (52.4)	128 (80.5)	51 (34.0)	<0.001
Biopsies taken prior to ESD*	164 (37.9)	42 (33.9)	64 (40.3)	58 (38.7)	0.53
*Numbers of missing values per center are shown in **Supplementary Table 7** Values are n (%) unless otherwise defined. CRC, colorectal cancer; ESD, endoscopic submucosal dissection; SD, standard deviation.					

**Table:** **Table 2** ESD-related outcomes (n=435 procedures)

	All centers n=435	Center #1 n=124	Center #2 n=159	Center #3 n=152	P value
En bloc resection	402 (92.4)	112 (90.3)	149 (93.7)	141 (92.8)	0.55
Conversion to piecemeal resection	33	12	10	11	
Scheduled ESD time, mean (SD), minutes*	136.8 (52.6)	176.8 (62.6)	108.1 (30.0)	134.7 (40.6)	<0.001
Procedure duration, mean (SD), minutes*	139.0 (95.4)	176.7 (106.3)	96.4 (77.8)	142.4 (86.0)	<0.001
Endoscopic maneuverability (as evaluated during ESD)*					<0.001
Good	49 (23.1)	10 (35.7)	24 (24.7)	15 (17.2)	
Average	79 (37.3)	0 (0.0)	45 (46.4)	34 (39.1)	
Poor	84 (39.6)	18 (64.3)	28 (28.9)	38 (43.7)	
Immediate perforation	38 (8.7)	14 (11.3)	16 (10.1)	8 (5.3)	0.16
Delayed perforation	10 (2.3)	1 (0.8)	8 (5.0)	1 (0.7)	0.015
Perforations requiring surgery	8 (1.8)	1 (0.8)	6 (3.8)	1 (0.7)	0.074
Hospitalization	264 (60.7)	96 (77.4)	22 (13.8)	146 (96.1)	<0.001
Duration of hospitalization, median (range), days*	1 (1–55)	1 (1–55)	1 (1–40)	1 (1–4)	<0.001
Adverse event during hospitalization*	21	10	3	8	
Readmission within 30 days after discharge*	33 (7.6)	10 (8.1)	20 (12.6)	3 (2.0)	0.002
*Numbers of missing values per center are shown in Supplementary Table 7 Values are n (%) unless otherwise defined. ESD, endoscopic submucosal dissection; SD, standard deviation.					

**Table:** **Table 3** Outcomes of all en bloc ESDs (n=402 procedures).

	All centers n=402	Center #1 n=112	Center #2 n=149	Center #3 n=141	P value
R0 resection*	236 (68.8)	70 (63.1)	63 (69.2)	103 (73.1)	0.24
R0 lateral margin*	251 (73.4)	80 (72.1)	65 (71.4)	106 (75.7)	0.72
R0 vertical margin*	272 (87.7)	94 (85.5)	58 (90.6)	120 (88.2)	0.59
Histology*					<0.001
Low-grade dysplasia	174 (43.3)	55 (55.6)	51 (34.2)	68 (52.3)	
High-grade dysplasia	76 (18.9)	14 (14.1)	22 (14.8)	40 (30.8)	
Invasive cancer	145 (36.1)	43 (38.4)	70 (47.0)	32 (22.7)	
Superficial submucosal invasion (<1000 μm)	41	12	18	11	
≥Deep submucosal invasion	80	20	47	13	
R0 resection with missing invasion depth	24	11	5	8	
Other ^†^	5 (1.2)	0 (0.0)	4 (2.7)	1 (0.7)	
Indication for additional surgical resection*	87 (21.8)	24 (21.4)	48 (32.7)	15 (10.6)	<0.001
FU-duration in months, mean (SD)*	20.9 (20.1)	20.6 (17.2)	23.5 (25.0)	19.0 (18.7)	0.39
Recurrence*	4 (1.6)	1 (0.9)	3 (4.1)	0 (0.0)	0.10
*Numbers of missing values per center are shown in **Supplementary Table 7** †Sessile serrated lesion without dysplasia (n=2 from center #2), inflammatory polyp/infiltrate without dysplasia (n=2 from center #2 and n=1 from center #3) Values are n (%) unless otherwise defined. FU, follow-up; SD, standard deviation.					

### Performance of current time scheduling practice

In complete case analysis (n=367), 257 ESDs (70%) were completed within ±1 hour of the scheduled time, 60 ESDs (16%) >1 hour ahead of the scheduled time, and 50 (14%) exceeded the scheduled time >1 hour. Explanations for completion >1 hour ahead of the scheduled time were described in none of the endoscopy reports. For the 50 ESDs in which the scheduled time was exceeded by >1 hour, explanations could be found in seven cases (submucosal fibrosis n=3, many intraprocedural bleedings n=2, poor overview during dissection n=2; **Supplementary Table 2** ). ESDs exceeding the scheduled time >1 hour were more likely to be converted to piecemeal resection (13/50 vs. 14/317; *P* <0.001) and also had a lower R0 resection rate (19/49 vs. 165/280; *P* =0.012). However, the rates of perforation (immediate: 5/50 vs. 22/317; *P* =0.39; delayed: 2/50 vs. 8/317; *P* =0.63), adverse events during hospitalization (4/38 vs. 13/201; *P* =0.32), and readmissions (5/49 vs. 22/317; *P* =0.38) were not significantly different.

**Fig. 1:**
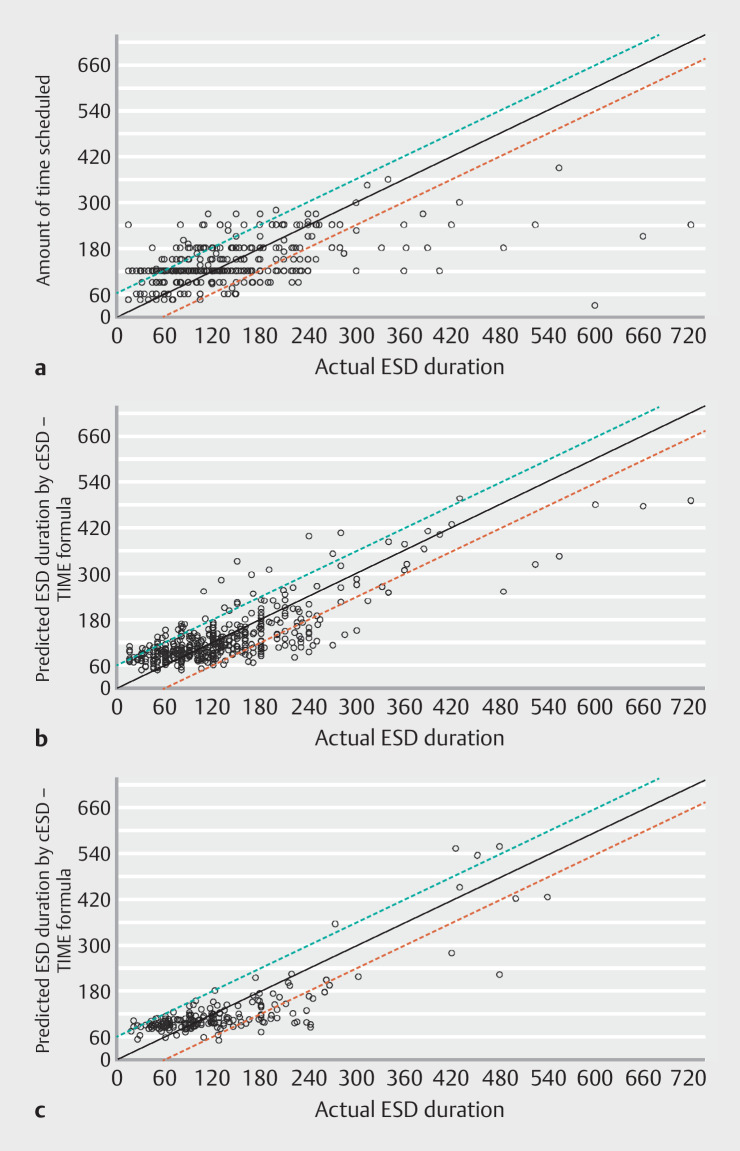
**a** Performance of current ESD planning practice in the analysis cohort (n=435 procedures). **b** Performance of the cESD-TIME formula in the analysis cohort (n=435 procedures) **c** Performance of the cESD-TIME formula in the Swedish validation cohort (n=199 procedures) after recalibration of the slope (β=0.8). The reference line “scheduled time=actual duration” is shown in the graph with ± 1 hour margin (green dashed line: running ahead >1 hour of the allotted time, red dashed line: exceeding the allotted time by >1 hour). ESD, endoscopic submucosal dissection.

The explained variance in ESD durations by current time planning practice was 27% (95% confidence interval [CI] 20%–35%; Fig. 1 **a** ). The proportions of procedures completed ±1 hour of the scheduled time (70%), >1 hour ahead of the scheduled time (17%) and exceeding the scheduled time >1 hour (13%) were comparable between complete case (n=367) and multiple imputation analyses (n=435).

### Performance of a previous Eastern prediction model

A prediction model for colorectal ESD duration (i.e. completion <60 minutes yes/no) was previously developed by Eastern endoscopists 6 , with a c-statistic of 0.70 in the development cohort and 0.69 in an Eastern external validation cohort. We evaluated the performance of this Eastern model in our analysis cohort and found a c-statistic of 0.70 (95% CI 0.62–0.77; **Supplementary Fig.2** ). Notably, the proportion of ESDs completed <60 minutes was much lower in our Western cohort (14%) as compared to the Eastern cohort (54%).

When changing the dichotomization cut-off over the entire range of ESD durations (15–720 minutes) in our analysis cohort, we found that both the proportion of ESDs completed within the cut-off time and the c-statistic proportionally increased with the cut-off value ( **Supplementary Fig. 3a, Supplementary Fig. 3b, Supplementary Fig. 3c** ). For example, when dichotomizing ESD duration into completion <120 minutes yes/no, the c-statistic was 0.75 (95% CI 0.69–0.80) and the proportion of ESDs completed <120 minutes was 51.0%. When using a dichotomization cut-off of 180 or 240 minutes, the c-statistic was 0.79 (95% CI 0.72–0.84) or 0.85 (95% CI 0.75–0.91) and the proportion of ESDs completed within the cut-off time was 77% or 91%, respectively.

### cESD-TIME formula development

Next, we developed a prediction model (the cESD-TIME formula) that included ESD duration as continuous outcome. A transformed version of the Eastern model yielded an R ^2^ of 61% ( **Supplementary Table 3** and **Supplementary Results** ). To evaluate whether the model could be simplified or its performance further improved, we applied regression-based backward selection with the four variables of the Eastern model and other pre-procedural factors that are associated with submucosal fibrosis or ESD experience (unadjusted associations between all candidate predictors and the outcome in **Supplementary Table 4** ). This resulted in the cESD-TIME formula, a six-variable model that includes tumor size, luminal circumference, morphology (as defined in the Eastern model 6 ), depressed area, inflammatory bowel disease, and consecutive number of colorectal ESDs performed ( Table 4 ). Notably, tumor location (as defined in the Eastern model or categorized into rectum and left and right hemicolon) was not selected in the final cESD-TIME formula, as well as the type of lesion (naïve or recurrent lesion to be resected), whether or not prior biopsies were taken and whether or not invasive cancer was suspected ( **Supplementary Table 5** ). The final cESD-TIME formula explained 63% of the variance in ESD duration (95% CI 57%–69%; Fig. 1 **b** ), with 79% of ESDs completed ±1 hour of the predicted time, 12% completed >1 hour ahead of the predicted time and 9.2% exceeding the predicted time >1 hour. An example calculation in the online calculator ( https://cesdtimeformula.shinyapps.io/calculator/ ) is provided in **Supplementary Fig. 4** .

**Table:** **Table 4** cESD-TIME formula to predict ESD duration in minutes.

Predictor	Definition/transformation	Beta	*P* value
Tumor size in mm	Size ^2^	0.018	<0.001
Luminal circumference in %	If ≤ 25%: count circumference as 0 If >25%: circumference ^2^	0.028	<0.001
Morphology	According to Li et al. GIE 2021: LST-NG=1	22.42	0.0035
Depressed area	Paris IIc component present=1, absent=0	–16.16	0.010
Inflammatory bowel disease	Present=1, absent=0	18.59	0.18
Consecutive number of colorectal ESD for endoscopist performing the procedure	If <130: use consecutive number without transformations If ≥ 130: count number as 130	–0.23	<0.001
Intercept		91.10	
ESD, endoscopic submucosal dissection; LST-NG, laterally spreading tumor with a non-granular surface pattern.			

The exploratory analyses with endoscopic maneuverability as potential predictor are detailed in the **Supplementary Results** .

### cESD-TIME formula validation

The internal and internal-external cross-validation procedures are shown in the **Supplementary Results** . The independent Swedish validation cohort consisted of 199 colorectal ESDs, performed by two endoscopists (#7: n=109, #8: n=90). Key tumor and ESD characteristics are shown in **Supplementary Table 6** . The mean lesion size was 43 mm (SD 28), and the mean procedure duration was 128 minutes (SD 92; 17% of procedures completed <60 minutes). The en bloc and R0 resection rate were 100% and 93% (186/199), respectively.

In its original form, the cESD-TIME formula explained 52% (range over 10 imputations: 51%–52%) of the variance in ESD duration in the Swedish cohort. Linear regression revealed moderate calibration (intercept: 5.3, slope: 0.78). Visual inspection of the scatter plot ( **Supplementary Fig. 5** ) suggested some overprediction of ESD duration. Given the differences in performance between the Dutch development and Swedish validation cohort (e.g. en bloc resection rate 92% vs. 100%, R0 resection rate 69% vs. 93%, mean ESD duration 128 vs. 139 minutes with a mean tumor size of 43 vs. 39 mm), we tested whether recalibration could improve the accuracy of the model’s predictions ( **Supplementary Results** ). Recalibration of the slope by multiplying all the predicted values by 0.8, the optimal value, yielded an R ^2^ of 66% (range over 10 imputations: 66%–67%; Fig. 1 **c** ). Based on these findings, we included a customizable slope for the linear predictor in the online calculator. As users acquire more information about their ESD performance level, the recalibration slope can be adjusted if necessary.

## Discussion

This study provides two useful tools for predicting colorectal ESD duration at Western endoscopy centers. First, we successfully validated a previous Eastern model 6 for ESD duration in a large Dutch cohort. However, this model had a dichotomized outcome and turned out to have more discriminative power when using higher dichotomization cut-offs. Second, we developed the new, easy-to-use cESD-TIME formula ( https://cesdtimeformula.shinyapps.io/calculator/ ), which predicted procedure duration as a continuous outcome. This formula increased the current planning accuracy more than two-fold (R ^2^ : 61% vs. 27%) and performed well (R ^2^ =66%) in a cohort with relatively higher ESD performance levels after recalibration. These prediction tools could facilitate individualized time scheduling of colorectal ESD at Western centers.

To our knowledge, this is the first study to provide empirical data on time planning outcomes of colorectal ESDs in Western practice, where logistical issues concerning ESD implementation stand out more strikingly due to a lack of experience and ESD experts 7 . This is illustrated by our finding that current unstandardized time planning was inaccurate, with a substantial proportion (~30%) of procedures running ahead or exceeding the allotted time. Exceeding more than 1 hour was also associated with significantly lower en bloc and R0 resection rates, which could probably be attributed to the increased fatigue and time pressure experienced by the endoscopist. Reasons for ESD completion ahead of or behind the scheduled time were rarely reported. Only for a few procedures that were completed >1 hour later could we deduce that unanticipated difficulties (submucosal fibrosis, intraprocedural bleedings) were the main causes of time being exceeded. It should be kept in mind that besides unanticipated peri-procedural events or findings, suboptimal time allocation may also explain why a procedure was completed ahead of or behind the scheduled time. To illustrate this, most ESDs in center #2 (73%) appeared to be scheduled for a standard 2-hour time slot, but a substantial proportion of these procedures turned out not to require 2 hours (120±0min: 9.3%, 120±30min: 25%, 120±60min: 64%). Altogether, our findings emphasize the necessity of systematic and more accurate time planning for colorectal ESD.

To improve planning accuracy, we first evaluated the performance of a previously published Eastern prediction model for colorectal ESD duration 6 . Interestingly, the Eastern prediction model was also valid in a Western setting, given the similar c-statistic values (0.70) in our cohort and the original development cohort. This may have come as a surprise, considering the large difference in performance levels between Eastern and Western ESD practice 2 28 . It appears that there may be certain “universal” pre-procedural factors that determine ESD duration. However, despite successful external validation, the Eastern model was still of limited use for Western practice because of the relatively low proportion of procedures completed within the 60-minute cut-off (14%). When increasing this cut-off value, both the c-statistic and the proportion of procedures completed within the cut-off time increased considerably. This indicates that the Eastern model seems more useful for determining whether an ESD is very likely or unlikely to take much time in Western ESD practice.

We decided not to further update the Eastern model, as the dichotomized outcome does not provide information on how much longer or shorter a procedure would take than the cut-off time. Instead, we developed a new formula that predicted ESD duration as continuous outcome. Using the four predictors from the Eastern model and some simple transformations, we were already able to construct a model with satisfactory performance. Strikingly, a relatively low β (+2.2 minutes) and high *P* value ( *P* =0.52) were found for tumor location, and this remained so after including different locations as separate variables or redefining the categories. Besides, tumor location was also omitted from the final cESD-TIME formula after backward selection, suggesting that the influence of this factor on ESD duration was limited. This was unexpected because anatomical location, in general, is closely correlated with endoscopic maneuverability, a crucial determinant of ESD difficulty 7 26 . However, a recent editorial has already pointed out that performing ESD in certain situations (e.g. patients with severe abdominal adhesions or a long and flexible colon) is always difficult, regardless of tumor location 7 . Besides, selection bias of lesions in supposedly difficult locations may also explain the limited predictive value of anatomical location. In any case, it appears that location does not always faithfully recapitulate endoscopic maneuverability, thus the complexity and duration of colorectal ESD.

Despite the superior performance of the cESD-TIME formula over current time planning practice, predicting exact procedure times remains quite challenging. For instance, this is reflected in the wide prediction intervals and the need for recalibration in the Swedish validation cohort, which was reasonable given the between-cohort difference in ESD performance. Although achieving 100% accuracy is probably utopian 29 , as we can learn from prediction modeling studies for surgical procedure times (highest R ^2^ values around 80% 30 31 32 33 34 ), we acknowledge that there is still room for improvement. As proposed by several ESD experts 7 26 , endoscopic maneuverability could be key to increasing the predictive performance. This is substantiated by a Japanese study 27 and our finding that maneuverability (as subjectively evaluated during the ESD) was a significant predictor of procedure duration ( **Supplementary Results** ). Unfortunately, no objective and standardized criteria are currently available for assessing endoscopic maneuverability. Jacques et al. recently proposed the Size, Maneuverability, Site, History (SMSH) score 35 , the “ESD-equivalent” of the SMSA-classification, for predicting R0 resection without perforation. However, we think that the Maneuverability category of this classification lacks sufficient detail for prediction of ESD duration, as it categorizes maneuverability only into “good” and “bad” without further specifications in the scoring criteria. Therefore, we propose some objective and more precise criteria for systematically classifying endoscopic maneuverability for colorectal ESD.

### Proposed assessment criteria for classifying endoscopic maneuverability for colorectal ESD

Endoscopic field of vision

1. Is it easy to make an overview picture of the lesion?
2. Is the lesion located behind a bowel fold?
3. Is the lesion only accessible in retroversion?

Approach for resection

1. Is it easy to touch the full margin of the lesion with the tip of the endoscope without changing the position of the patient or assistance of a cap?
2. Is it easy to obtain and maintain a non-perpendicular view of the lesion?
3. ESD, endoscopic submucosal dissection.

These criteria can be integrated in a classification system with specific weighting factors or multiplication coefficients. Of course, such a system should preferably be developed by a large expert panel of Western and Eastern endoscopists.

While awaiting further improvements in and validation of the abovementioned ESD time prediction models, we suggest the following tips for time scheduling in current Western ESD practice. As a first screening, we recommend using the Eastern model to determine which procedures are very likely or unlikely to be completed within 120 minutes. The 120-minute cut-off is proposed because in this case the lowest (“Easy”) and highest (“Very difficult”) categories both have a >80% chance of ESD completion within and outside the cut-off time, respectively. Thus, “Easy” procedures should be scheduled for less than 2 hours, and “Very difficult” procedures for 2 hours at least. To determine how much longer or shorter a procedure would take than the cut-off time, and for the “Intermediate” or “Difficult” procedures, we recommend using the cESD-TIME formula as main guidance. In our opinion, the predicted duration from the formula can be used to determine the optimal amount of scheduled time.

The main limitations of our study are related to the retrospective design. As a result, some relevant data (e.g. reasoning behind current time scheduling practice, size of the margin of normal tissue taken along for each ESD) and potentially important pre-procedural predictors (e.g. flexibility of the colon, maneuverability-related factors mentioned in list above were unavailable for analysis. The latter issue particularly impacted the clinical value of the cESD-TIME formula, as we were only able to develop a formula with moderate performance (R ^2^ of ~60%) based on the data that were available. Moreover, it was unclear how accurate certain variables were assessed that can be prone to interobserver variability (e.g. tumor size, circumference). This may have decreased the model’s predictive accuracy but increased its generalizability as our data reflect daily practice in which inaccurate assessments may sometimes occur. Third, the findings from our study may not be fully applicable to right-sided lesions due to the relatively small proportion of such lesions in our cohort (7.8%). However, it is important to note that these lesions typically are not recommended as initial cases for novice ESD endoscopists (the primary target audience of the cESD-TIME formula) because the risk of perforation during right-sided ESD in European practice can be relatively high (up to ~20%) 22 . Lastly, no traction device-assisted ESD techniques were used in our cohort. This is relevant because these techniques may markedly change the nature of a procedure 26 , and thereby also the factors determining ESD duration. Therefore, our findings may not be valid for traction device-assisted ESD procedures.

## Conclusions

To conclude, by validating a previous Eastern prediction model and developing the new cESD-TIME formula, we established two useful prediction tools that could aid time scheduling of colorectal ESDs in Western settings. These tools have the potential to considerably improve time planning accuracy, as well as facilitating more widespread implementation of colorectal ESD at Western endoscopy centers.

## Supporting information

Supplementary material Zusatzmaterial
