# Correction: Predicting procedure duration of colorectal endoscopic submucosal dissection at Western endoscopy centers

**DOI:** 10.1055/a-2150-5897

**Published:** 2023-08-10

**Authors:** Hao Dang, Nik Dekkers, Ewout W. Steyerberg, Francisco Baldaque-Silva, Masami Omae, Krijn J.C. Haasnoot, Laurelle van Tilburg, Kate Nobbenhuis, Jolein van der Kraan, Alexandra M.J. Langers, Jeanin E. van Hooft, Wilmar de Graaf, Arjun D. Koch, Paul Didden, Leon M.G. Moons, James C.H. Hardwick, Jurjen J. Boonstra

**Affiliations:** 1 4501 Gastroenterology and Hepatology, Leiden University Medical Center, Leiden, Netherlands; 2 4501 Biomedical Data Sciences, Leiden University Medical Center, Leiden, Netherlands; 3 59562 Endoscopy Unit, Center for Upper Digestive Diseases, Karolinska Hospital, Stockholm, Sweden; 4 37824 Advanced Endoscopy Center Carlos Moreira da Silva, Pedro Hispano Hospital, Matosinhos, Portugal; 5 8124 Gastroenterology & Hepatology, University Medical Centre Utrecht, Utrecht, Netherlands; 6 6993 Gastroenterology and Hepatology, Erasmus MC, Rotterdam, Netherlands; 7 8124 Gastroenterology and Hepatology, University Medical Centre Utrecht, Utrecht, Netherlands



In the above-mentioned article an author's name was corrected. This was corrected in the online version on 10.08.2023.

