# Supplementary material for: Predicting procedure duration of colorectal endoscopic submucosal dissection at Western endoscopy centers
Source: Endosc Int Open. 2023 Aug 7;11(8):E724–32. doi: 10.1055/a-2122-0419 (PMC10629487; doi:10.1055/a-2122-0419)
Supplement: Supplementary file 1 — Supplementary material [file 10-1055-a-2122-0419_21267201.pdf]

Supplementary material

Supplementary Methods

Clinical parameters and definitions

The following parameters were retrospectively retrieved from electronical medical records: baseline patient characteristics (age, sex, body mass index (BMI), comorbidities, use of anticoagulant therapy) tumor characteristics (location, morphology, size, luminal circumference, recurrent or naïve lesion to be resected, optical diagnosis, prior biopsies and histology), treatment characteristics (amount of time scheduled, actual procedure duration, endoscopic maneuverability, consecutive number of ESD performed, conversion to piecemeal resection) and clinical outcomes after ESD (adverse events, hospitalizations, readmissions and reinterventions within 30 days after discharge, additional surgical treatment, follow-up). Some parameters were not routinely recorded in medical records and endoscopy reports of the different centers.

Patient comorbidity was quantified using the Charlson Comorbidity Index[1] and the American Society of Anesthesiologists physical status classification system. Optical diagnosis of colorectal tumors was performed according to guideline recommendations[2] (see **Supplementary Fig.6** for endoscopic images of lesions with suspected invasive cancer). Tumor size was defined as the largest diameter of the lesion as determined during endoscopic assessment. The left hemicolon was defined as the colon between the splenic flexure and rectosigmoid junction, and the right hemicolon was defined as the colon proximal to the splenic flexure. A depression was defined as a sunken place on the surface of the lesion, relative to the surrounding polyp tissue or normal mucosa (**Supplementary Fig.6**). *En bloc* resection was defined as a tumor excision in one piece without fragmentation. Histologically complete resections (R0) were defined as *en bloc* resection with negative horizontal and vertical resection margins. Perforations were classified as immediate (detected during the procedure) or delayed (detected after the procedure). Additional surgery after ESD was indicated for submucosally invasive carcinomas with ≥1 histological high-risk feature[3]: incomplete resection, lymphovascular invasion, poor differentiation, deep submucosal invasion (excluded as high-risk feature in the last few years[4]) and high-grade tumor budding (included as high-risk feature after scoring standardization[5]). In general, follow-up was performed according to the Dutch surveillance guidelines[6, 7].

Only explicit mentions of parameters were recorded. In case a certain parameter was missing, we classified it as such and did not consider it to be “absent”, “negative” or equivalent. Unless there were clear signs of missingness not at random (MNAR; e.g. certain categories are completely missing, only extreme values are being reported), we assumed that missing values were not MNAR.

Supplementary material

ESD procedure

In the Dutch ESD cohort, all assessments and ESDs were performed by 6 experienced interventional endoscopists (WdG, ADK, PD, LMG, JCH, JJB) who followed extensive training in ESD (tutorial courses and animal *in vivo* training). None of the endoscopists had prior experience in ESD before. In the early phase of the study period (2011 till ~2014-2015), ESD was performed according to the conventional method as described previously[8]. Thereafter, ESD was mostly carried out according to the pocket-creation[9] or tunneling method[10]. The perimeter of the lesion was not routinely marked before starting the dissection. The adopted ESD method was not routinely recorded in the endoscopy reports.

In the Swedish validation cohort, all ESDs were performed by 2 experienced interventional endoscopists (FBS, MO) who have followed extensive training in ESD (tutorial courses and animal *in vivo* training). Both endoscopists had limited prior experience in ESD before their first procedure in this series.

Variable definitions of the previously developed Eastern prediction model

The Eastern prediction model for colorectal ESD duration[11] consisted of 4 parameters, each assigned with a certain number of points based on the β-coefficient in multivariable analysis (1. Tumor size: 30-50 mm, 1 point, ≥50 mm, 2 points; 2. Circumference: more than two-third, 2 points; 3. Location: cecum or dentate line, 1 point, flexure, 2 points; 4. Morphology: non-granular laterally spreading tumor, 1 point). The total sum of points is then categorized into “easy” (0), “intermediate” (1), “difficult” (2-3) and “very difficult” (≥4).

Predicted probabilities could not be calculated because the intercept of the Eastern prediction model was not reported in the original paper. Therefore, calibration of the model also could not be evaluated by a calibration plot (which presents the predicted probability against the observed risk).

Statistical analyses

All statistical analyses were performed using R v4.1.2. Nominal and ordinal variables were expressed as frequencies and percentages, and continuous variables as means and standard deviations (SD). Pearson’s chi-square test was used to compare categorical data. Continuous variables were compared using a one-way analysis of variance. A p-value of <0.05 was considered statistically significant.

Multiple imputation by chained equations (*mice* package, 10 datasets) was used to address missing data while respecting the correlation structure. Details on the extent of missing data per variable are provided in **Supplementary Table 7**.

Supplementary material

After imputation, the performance of current time planning practice was quantified by calculating the proportion of explained variance (R^2). Pooling of the R^2 was done according to Rubin's Rules[12] using the *pool.r.squared* function. The performance of the Eastern prediction model for ESD duration[11] was quantified by calculating the c-statistic. The c-statistic of the 10 different datasets were calculated using the *ROCit* package and pooled according to Rubin's Rules[12] using the *pool_auc* function. Visualizations of the receiver operating characteristic (ROC) and probabilities per scoring category were created from a stochastic single imputation dataset (the first of a series of datasets generated through multiple imputation).

For the development of the cESD-TIME formula, no formal sample size calculation was performed. This was because the number of subjects per predictor (SPP) was *a priori* expected to be much larger (>20-30 SPP) than rules of thumb (2 SPP) which have been proposed for adequate estimation of regression coefficients[13, 14].

The cESD-TIME formula was developed using the *rms* package. Continuous variables were winsorized at the 1st and 99th percentile before imputation[15]. The cESD-TIME formula was built using multivariable linear regression with backward selection based on $p<0.20$[15]. Uniform shrinkage of regression coefficients was applied when the mean bootstrapped shrinkage factor was <0.99[15]. Non-linearity of continuous predictors was assessed using polynomials, variable restrictions and transformations, and restricted cubic splines. R^2 was used as the outcome measure for model performance. Internal bootstrap validation (*validate* function, 1000 replications) was used to evaluate the risk of overfitting. Internal-external cross-validation by omission of each of the 3 centers in turn[16] was used to mimic the situation that the model is applied in a new center. This cross-validation strategy was also performed with omission of each of the ESD endoscopists in turn. The final model was based on the full analysis cohort[16] and was assessed for overall performance and calibration in fully external validation. If the R^2 of the developed model in external validation were lower than the optimism-corrected R^2, simple recalibration methods such as calibration-in-the-large and slope recalibration of the linear predictor were considered first before using more advanced methods such as model revision and extension.

The online calculator was built using the *shiny* package, and Bayesian estimates and prediction intervals were created using the *brms* package and the *predict* function. All model parameters were set as non-informative priors.

Supplementary material

Supplementary Results

Transformations of a previous Eastern prediction model

Linear regression showed that the original Eastern model explained 45% of the variance of the ESD durations (95%-CI: 38-52%). After transformation of the dichotomized quantitative variables of the Eastern model to continuous variables and non-linear fitting of these variables, the model's performance increased to 61% (full model with regression coefficients shown in **Supplementary Table 3**). Including all locations from the Eastern model (cecum, dentate line, flexure) as 3 separate yes/no variables did not further increase the R^2 (61%, 95%-CI: 54-68%). Moreover, the performance remained unchanged when categorizing tumor location into rectum, left and right hemicolon (R^2=61%, 95%-CI: 54-67%). Lastly, ungrouping the combined item "morphology" into its 2 separate components (gross morphology: protruding, sessile or flat; granularity: granular or non-granular surface) also did not considerably improve the model's performance (R^2=62%, 95%-CI: 55-67%).

Endoscopic maneuverability as potential predictor in the cESD-TIME formula

Endoscopic maneuverability has been proposed as a crucial determinant of ESD complexity and duration[17-19]. In the analysis cohort, maneuverability was subjectively evaluated and reported in 49% of cases. After imputation and backward selection, maneuverability was included as predictor in the resulting model (β=19, p<0.001; **Supplementary Table 8**), together with the other 6 variables of the cESD-TIME formula. The R^2 of this exploratory model was 65% (95%-CI: 59-71%).

Internal validation and internal-external cross-validation of the cESD-TIME formula

Internal validation of the 6-variable cESD-TIME formula revealed a bootstrapped mean optimism of 1.7% (range over 10 imputations: 1.4-2.1%) in the R^2 estimates. Internal-external cross-validation showed that leaving out center #1 from model development followed by performance testing in #1 resulted in a R^2 of 67% (range over 10 imputations: 63-71%; **Supplementary Table 9**). Leaving out center #2 or #3 resulted in a R^2 of 57% (range over 10 imputations: 54-62%) and 54% (range over 10 imputations: 48-56%), respectively. When validated across the endoscopists in the Dutch cohort, the R^2 ranged between 43-70%.

Recalibration of the cESD-TIME formula in the Swedish validation cohort

Calibration-in-the-large by subtracting 30 minutes, the optimal value, from all the predicted values increased the R^2 to 61% (range over 10 imputations: 61-62%). Recalibration of the slope by multiplying all the predicted values by 0.8, the optimal value, yielded an R^2 of 66% (range over 10 imputations: 66-67%; **Fig.1C**). Additional calibration-in-the-large did not further increase the R^2 (66%, range over 10 imputations: 66-67%). Comparable results were found when validating the

Supplementary material

recalibrated model (β=0.8) for the endoscopists in the Swedish cohort individually (endoscopist #7: R^2=65%, range over 10 imputations: 64-66%; endoscopist #8: R^2=66%, range over 10 imputations: 66-67%).

Supplementary material

Supplementary Tables

Supplementary Table 1. Patient characteristics of the analysis cohort (n=433 patients)

	All centers n = 433 *	Center #1 n = 124	Center #2 n = 157 *	Center #3 n = 152	P-value
**Age**, mean (SD), years	67.3 (8.7)	66.8 (9.5)	66.6 (8.5)	68.4 (8.3)	0.16
**Sex**, male (%)	270 (62.4)	77 (62.1)	99 (63.1)	94 (61.8)	0.97
**BMI**, mean (SD), kg/m² **	26.8 (4.7)	26.5 (3.9)	27.6 (5.6)	26.3 (4.4)	**0.046**
**ASA score** **					**<0.001**
I	85 (19.7)	33 (26.6)	22 (14.2)	30 (19.7)	
II	299 (69.4)	86 (69.4)	101 (65.2)	112 (73.7)	
III or IV	47 (10.9)	5 (4.0)	32 (20.6)	10 (6.6)	
**Charlson Comorbidity Index**, mean (SD)	3.3 (1.9)	2.9 (1.8)	3.9 (2.2)	2.9 (1.6)	**<0.001**
**Use of anticoagulants**	112 (25.9)	33 (26.6)	49 (31.2)	30 (19.7)	0.069
Discontinued before ESD	55 (12.7)	17 (13.7)	24 (15.3)	14 (9.2)	0.25
**Inflammatory bowel disease**	15 (3.5)	4 (3.2)	8 (5.1)	3 (2.0)	0.32
**Presence of Lynch syndrome**	16 (3.7)	3 (2.4)	10 (6.4)	3 (2.0)	0.083
**Patient history of CRC**	18 (4.2)	8 (6.5)	9 (5.7)	1 (0.7)	**0.026**
**Family history of CRC** **	81 (18.8)	34 (27.4)	18 (11.5)	29 (19.1)	**0.003**

*The number of patients is lower than the number of procedures because two patients underwent 2 single ESDs at 2 different time points
Numbers of missing values per center are shown in **Supplementary Table 7
Values are n (%) unless otherwise defined.

ASA: American Society of Anesthesiologists physical status classification system, *BMI:* Body mass index, *CRC*: colorectal cancer, *ESD:* endoscopic submucosal dissection, *SD*: standard deviation

Supplementary material

Supplementary Table 2. Reasons for cESD completion >1 hour ahead of/behind the scheduled time in the analysis cohort (n=367 procedures, complete case analysis)

	N (%)
**>1 hour ahead of the scheduled time**	60 (16.3)
Reason unclear or not specified	60
**>1 hour behind the scheduled time**	50 (13.6)
Submucosal fibrosis	3
Many intraprocedural bleedings	2
Poor overview during dissection	2
Reason unclear or not specified	43

Supplementary Table 3. Transformed Eastern model to predict ESD duration in minutes

Predictor	Definition/transformation	Beta	P-value
**Tumor size in mm**	Size^2	0.019	<0.001
**Luminal circumference in %**	If ≤25%: count circumference as 0<br>If >25%: circumference^2	0.028	<0.001
**Location**	According to Li et al. GIE 2021:<br>cecum or dentate line = 1, flexure = 2	2.27	0.52
**Morphology**	According to Li et al. GIE 2021:<br>LST-NG = 1	18.80	0.0021
**Intercept**		70.07	

LST-NG: laterally spreading tumor with a non-granular surface pattern

Supplementary material

Supplementary Table 4. Associations between all candidate predictors and ESD duration in univariable linear regression

Predictor	Definition/transformation	Beta	P-value
**Tumor size in mm**	Size^2	0.026	<0.001
**Luminal circumference in %**	If ≤25%: count circumference as 0<br>If >25%: circumference^2	0.057	<0.001
**Tumor location**	According to Li et al. GIE 2021:<br>Dentate line or cecum = 1, flexure = 2	14.53	0.012
**Morphology**	According to Li et al. GIE 2021:<br>LST-NG = 1	-21.66	0.026
**Depressed area**	Paris IIc component present = 1	-55.98	<0.001
**Inflammatory bowel disease**	Present = 1, absent = 0	27.37	0.23
**Suspected invasive cancer**	Yes = 1, no = 0	-34.76	<0.001
**Prior biopsy taken**	Yes = 1, no = 0	10.23	0.27
**Type of lesion to be resected**	Naïve lesion = 1, recurrence = 2	-34.49	0.090
**Consecutive number of colorectal ESD for endoscopist performing the procedure**	If <130: use consecutive number without transformations<br>If ≥130: count number as 0	-0.22	0.024

ESD: endoscopic submucosal dissection, *LST-NG:* laterally spreading tumor with a non-granular surface pattern

Supplementary material

Supplementary Table 5. Associations between all candidate predictors and ESD duration in multivariable linear regression

Predictor	Definition/transformation	Beta	P-value
**Tumor size in mm**	Size^2	0.018	<0.001
**Luminal circumference in %**	If ≤25%: count circumference as 0<br>If >25%: circumference^2	0.028	<0.001
**Location**	According to Li et al. GIE 2021:<br>cecum or dentate line = 1, flexure = 2	2.24	0.52
**Morphology**	According to Li et al. GIE 2021:<br>LST-NG = 1	21.92	<0.001
**Depressed area**	Paris IIc component present = 1	-18.49	0.0046
**Inflammatory bowel disease**	Present = 1, absent = 0	19.66	0.16
**Suspected invasive cancer**	Yes = 1, no = 0	6.55	0.27
**Prior biopsy taken**	Yes = 1, no = 0	-2.55	0.65
**Type of lesion to be resected**	Naïve lesion = 1, recurrence = 2	-10.26	0.41
**Consecutive number of colorectal ESD for endoscopist performing the procedure**	If <130: use consecutive number without transformations<br>If ≥130: count number as 0	-0.23	<0.001
**Intercept**		88.59	

ESD: endoscopic submucosal dissection**,** *LST-NG:* laterally spreading tumor with a non-granular surface pattern

Supplementary material

Supplementary Table 6. Key tumor and ESD characteristics of the independent Swedish validation cohort

	**N (%)**
Tumor characteristics	
**Location**	
Right hemicolon	71 (35.7)
Left hemicolon	43 (21.6)
Rectum	85 (42.7)
**Recurrence to be resected**	7 (3.5)
**Gross morphology**	
Pedunculated	2 (1.0)
Sessile	192 (96.5)
Flat	5 (2.5)
**Presence of a depressed area**	17 (8.5)
**Non-granular surface**	9 (4.5)
**Lesion size**, mean (SD), mm *	42.8 (27.8)
**Luminal circumference**, mean (SD), % **	31.9 (18.8)
**Suspected CRC in lesion**	10 (5.0)
**Inflammatory bowel disease**	11 (5.5)
ESD performance indicators	
**Procedure duration,** mean (SD), minutes *	127.9 (91.9)
***En bloc* resection**	199 (100)
**R0 resection (among *en bloc* resections)**	186 (93.5)

*N=2 missing
**N=3 missing

ESD: endoscopic submucosal dissection, *SD:* standard deviation

Supplementary material

Supplementary Table 7. Number of missing values per variable

	All centers n = 435	Center #1 n = 124	Center #2 n = 159	Center #3 n = 152
Supplementary Table 1 *Patient characteristics of the analysis cohort*				
**BMI** *	35 (8.0)	6 (4.8)	25 (15.9)	4 (2.6)
**ASA score** *	2 (0.5)	0 (0.0)	2 (1.3)	0 (0.0)
**Family history of CRC** *	1 (0.2)	0 (0.0)	1 (0.6)	0 (0.0)
Table 1. *Lesion characteristics of the analysis cohort*				
**Location**				
Lesion extending to the dentate line **	6 (2.4)	0 (0.0)	4 (4.5)	2 (2.6)
Junction of the sigmoid and descending colon ***	53 (52.5)	1 (7.1)	35 (89.7)	17 (35.4)
**Gross morphology**	13 (2.9)	0 (0.0)	8 (5.0)	5 (3.3)
**Depression**	15 (3.4)	3 (2.4)	10 (6.3)	2 (1.3)
**Granularity**	230 (52.9)	94 (75.8)	115 (72.3)	21 (13.8)
**Tumor size**	17 (3.9)	0 (0.0)	14 (8.8)	3 (2.0)
**Luminal circumference**	178 (40.9)	15 (12.1)	118 (74.2)	45 (29.6)
**Suspected CRC in lesion**	2 (0.5)	0 (0.0)	0 (0.0)	2 (1.3)
**Biopsies taken prior to ESD**	2 (0.5)	0 (0.0)	0 (0.0)	2 (1.3)
Table 2. *ESD-related outcomes*				
**Scheduled ESD time**	4 (0.9)	2 (1.6)	0 (0.0)	2 (1.3)
**Procedure duration**	64 (14.7)	13 (10.5)	49 (30.8)	2 (1.3)
**Endoscopic maneuverability (as evaluated during ESD)**	223 (51.3)	96 (77.4)	62 (39.0)	65 (42.8)
**Duration of and complications during hospitalization**	2 (0.5)	0 (0.0)	1 (0.6)	1 (0.7)
**Readmission within 30 days after discharge**	1 (0.2)	0 (0.0)	0 (0.0)	1 (0.7)
Table 3. *Outcomes of all en bloc ESDs*				

Supplementary material

**R0 resection ******	59 (14.7)	1 (0.9)	58 (38.9)	0 (0.0)
R0 lateral margin ****	60 (14.9)	1 (0.9)	58 (38.9)	1 (0.7)
R0 vertical margin ****	92 (22.9)	2 (1.8)	85 (57.0)	5 (3.5)
**Histology and indication for additional surgery ******	2 (0.5)	0 (0.0)	2 (1.3)	0 (0.0)
**Follow-up duration ******	148 (36.8)	6 (5.4)	78 (52.3)	64 (45.4)
**Recurrence ******	145 (36.1)	5 (4.5)	76 (51.0)	64 (45.4)

*Percentages over n=433 patients (n=157 for center #2)
**Percentages over number of rectal lesions (n=251 total; #1: n=87, #2: n=88, #3: n=76)
***Percentages over number of lesions in the sigmoid or descending colon (n=101 total; #1: n=14, #2: n=39, #3: n=48)
**** Percentages over number of *en bloc* ESDs (n=402 total; #1: n=112, #2: n=149, #3: n=141)

ASA: American Society of Anesthesiologists physical status classification system, *BMI:* Body mass index, *CRC*: colorectal cancer, *ESD:* endoscopic submucosal dissection

Supplementary Table 8. cESD-TIME formula with endoscopic maneuverability to predict ESD duration in minutes

Predictor	Definition/transformation	Beta	P-value
**Tumor size in mm**	Size^2	0.018	<0.001
**Luminal circumference in %**	If ≤25%: count circumference as 0<br>If >25%: circumference^2	0.026	<0.001
**Morphology**	According to Li et al. GIE 2021:<br>LST-NG = 1	21.84	0.0035
**Depressed area**	Paris IIc component present = 1, absent = 0	-13.91	0.022
**Inflammatory bowel disease**	Present = 1, absent = 0	21.72	0.10
**Consecutive number of colorectal ESD for endoscopist performing the procedure**	If <130: use consecutive number without transformations<br>If ≥130: count number as 0	-0.20	<0.001
**Endoscopic maneuverability**	“Good” = 1, “Average” = 2, “Poor” = 3	18.82	<0.001
**Intercept**		47.94	

ESD: endoscopic submucosal dissection, *LST-NG:* laterally spreading tumor with a non-granular surface pattern

Supplementary material

Supplementary Table 9. Internal-external cross-validation

Omitted unit	R^2 of the prediction model (which was developed in the other units) in the omitted unit	R^2 range over 10 imputations
Center-based cross-validation		
**Center #1**	67.1	62.9 – 70.6
**Center #2**	57.3	53.6 – 62.4
**Center #3**	53.9	47.6 – 56.2
*Endoscopist-based cross-validation**		
**Endoscopist #1**	70.4	67.1 – 74.8
**Endoscopist #2**	66.5	60.8 – 70.1
**Endoscopist #3**	59.2	56.8 – 64.0
**Endoscopist #5**	55.7	47.9 – 58.1
**Endoscopist #6**	43.3	36.7 – 47.5

*Internal-external cross-validation was not performed for endoscopist #4 because the number of procedures was relatively low (n=8), which resulted in unreliable performance estimates.

Supplementary material

Supplementary Figures

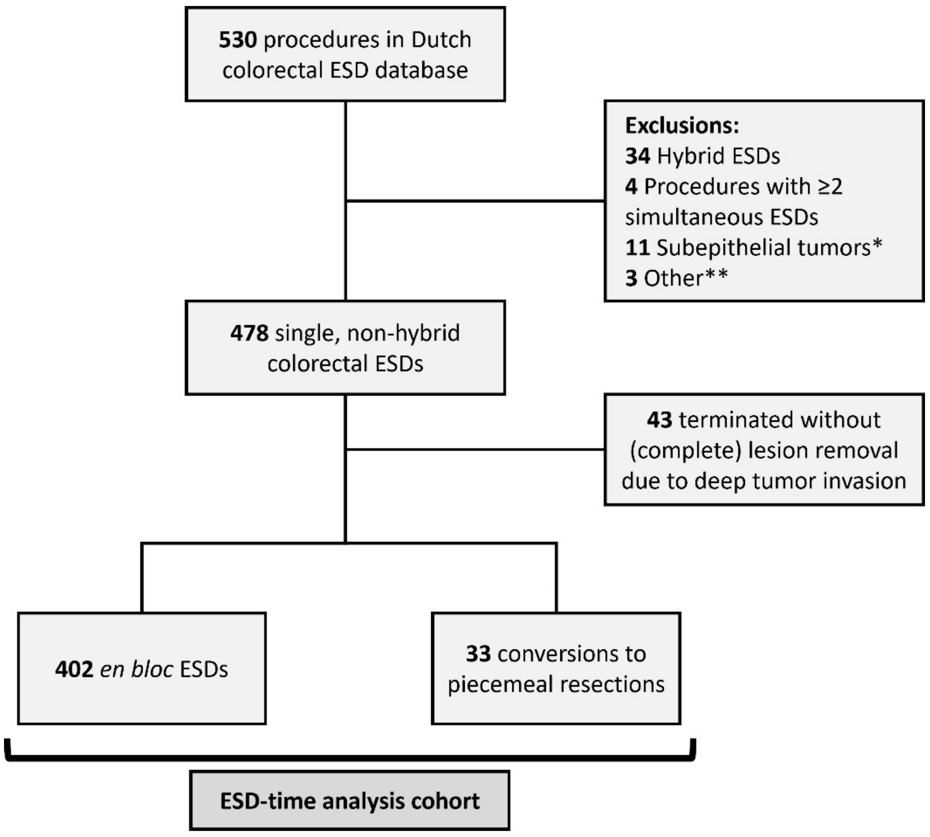

Supplementary Figure 1. Flowchart of patient inclusion.

*7 neuro-endocrine tumors, 2 gastrointestinal stroma cell tumors, 2 lipomas
**2 secondary scar resections, 1 anal intraepithelial neoplasia.

ESD: endoscopic submucosal dissection.

Supplementary material

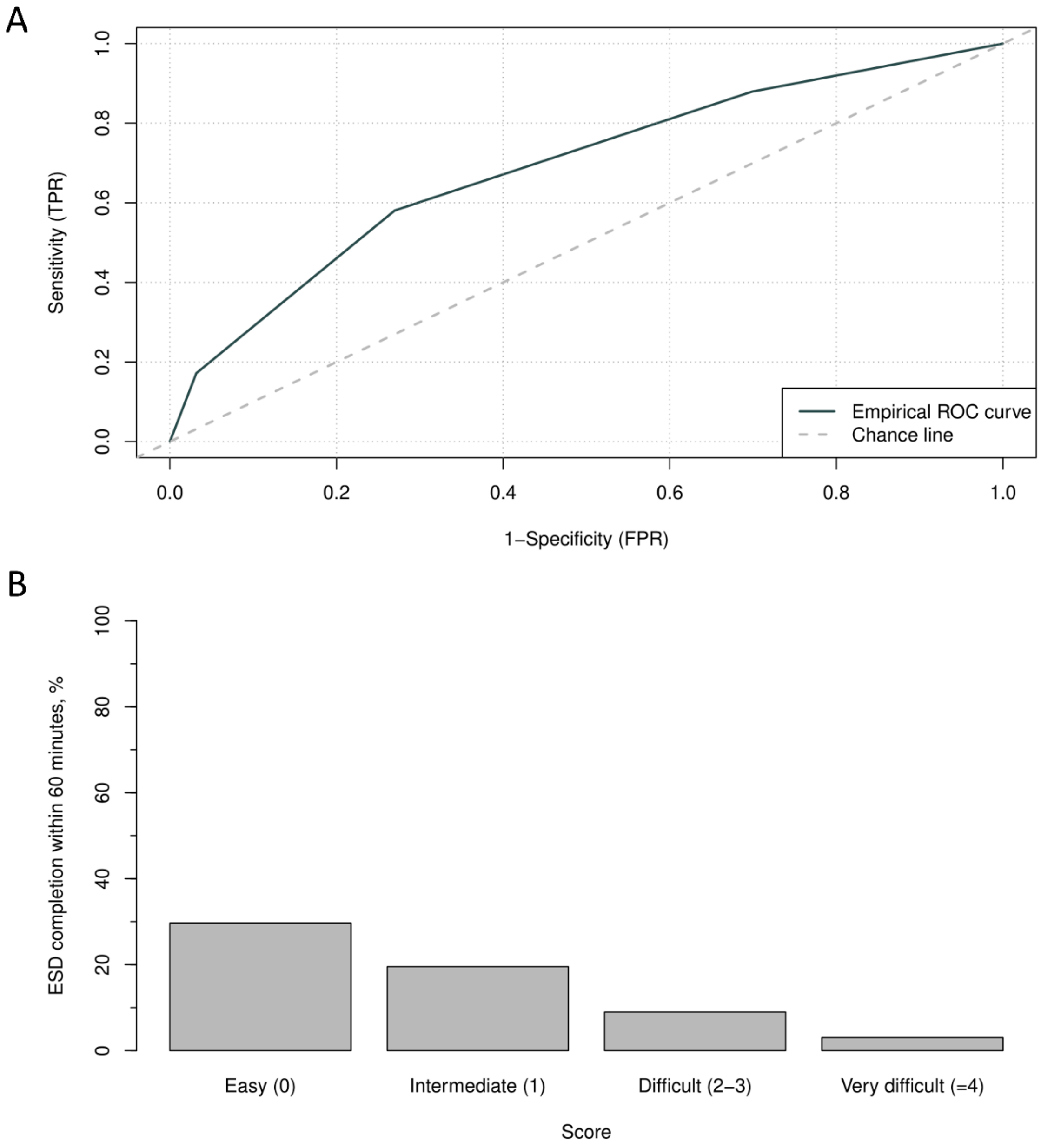

Supplementary Figure 2A. Receiver operating characteristic of the Eastern model in the analysis cohort (n=435 procedures; visualization using single imputation, calculation of the c-statistic using multiple imputation).

Supplementary Figure 2B. Proportions of ESD completion <60 minutes per scoring category as predicted by the Eastern model in the analysis cohort (n=435 procedures; visualization using single imputation, calculation of the c-statistic using multiple imputation).

ESD: endoscopic submucosal dissection, *FPR:* false positive rate, *ROC:* receiver operating characteristic, *TPR:* true positive rate.

Supplementary material

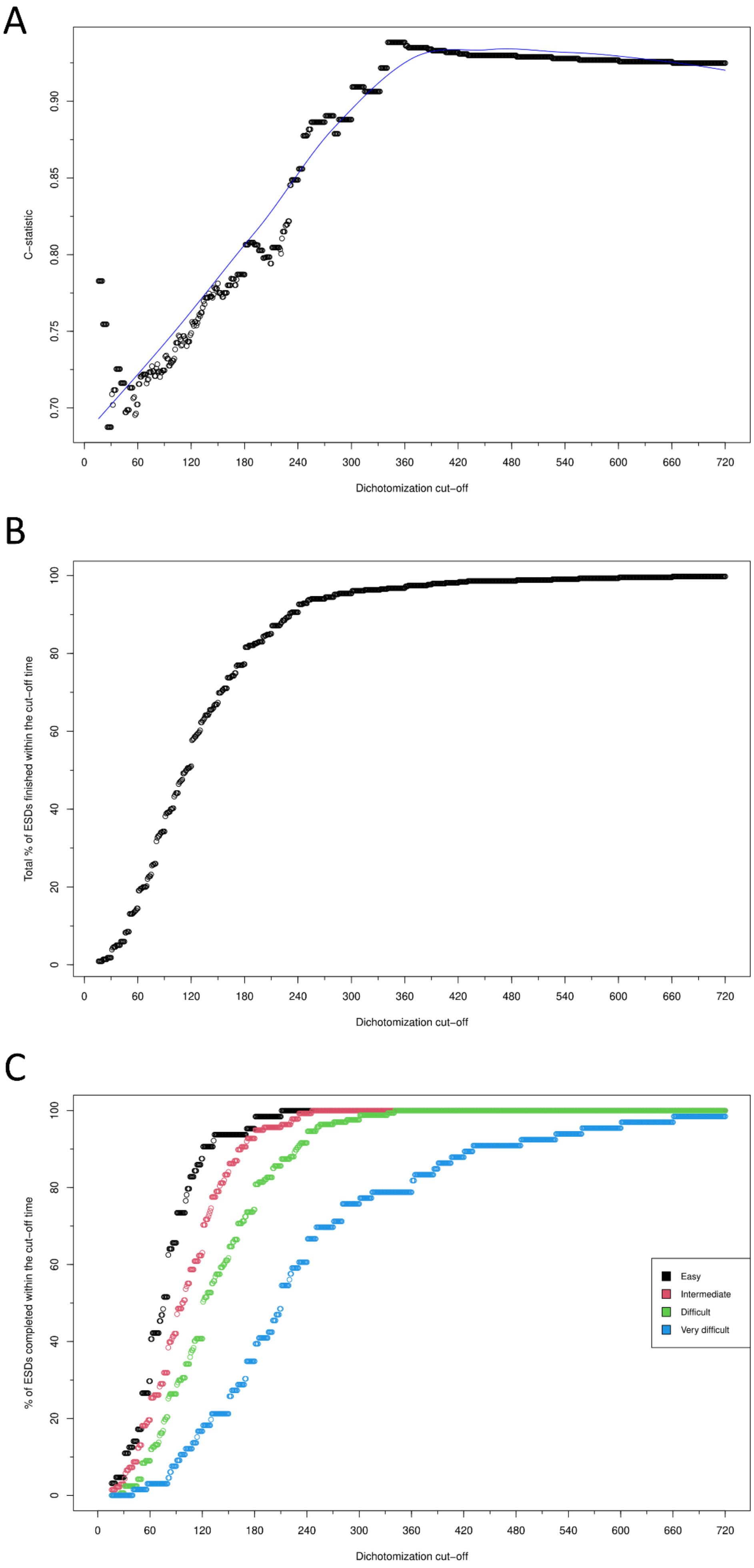

Supplementary material

Supplementary Figure 3A. C-statistic values for different dichotomization cut-offs of the Eastern model. The blue trend line is calculated using local polynomial regression fitting (*loess* function).

Supplementary Figure 3B. Total proportion of ESDs finished within the cut-off time for different dichotomization cut-offs of the Eastern model.

Supplementary Figure 3C. Total proportion of ESDs finished within the cut-off time per model category for different dichotomization cut-offs of the Eastern model.

ESD: endoscopic submucosal dissection

Supplementary material

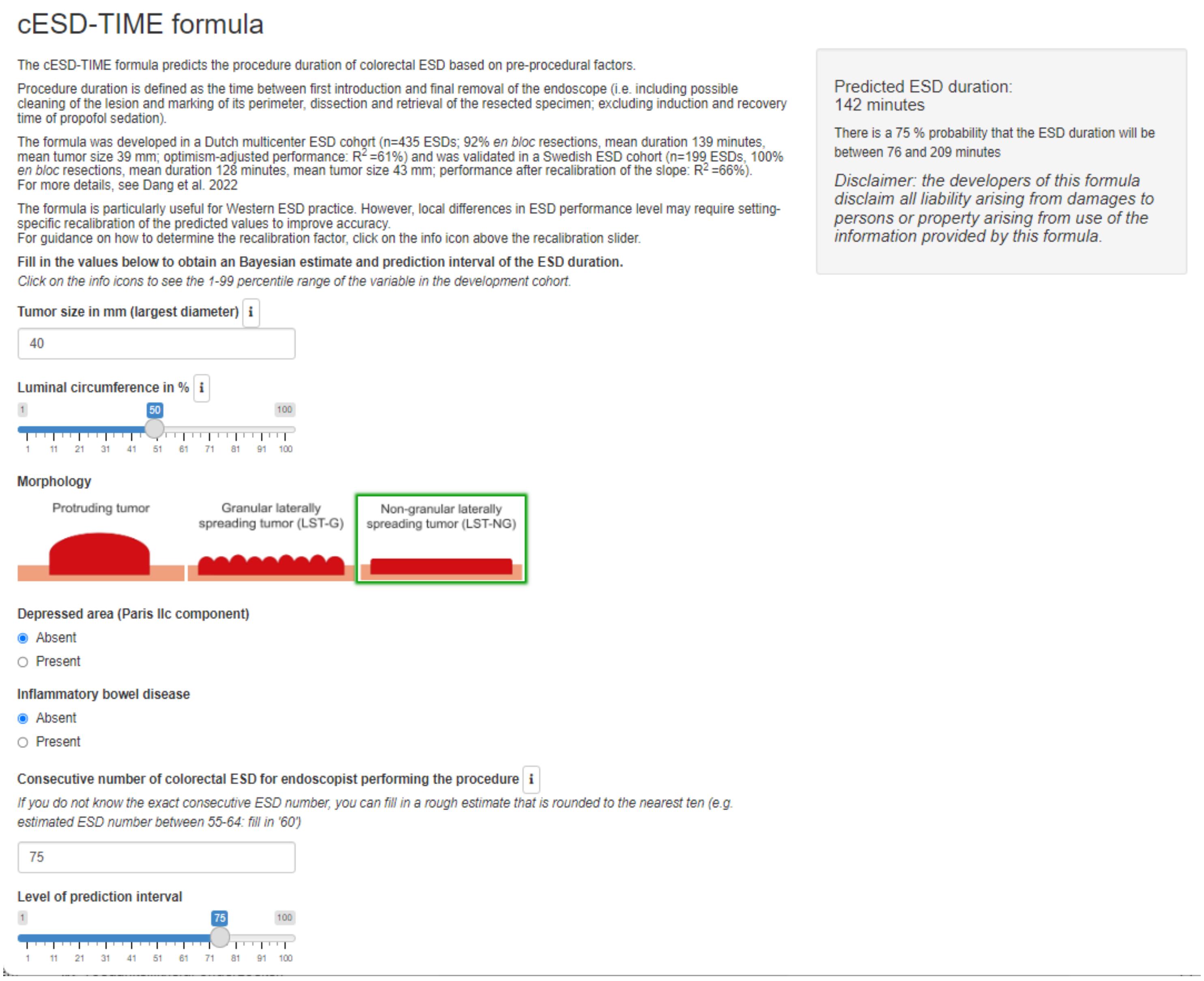

Supplementary Figure 4. Example calculation with the cESD-TIME formula in the online calculator (https://cesdtimeformula.shinyapps.io/calculator/).

Supplementary material

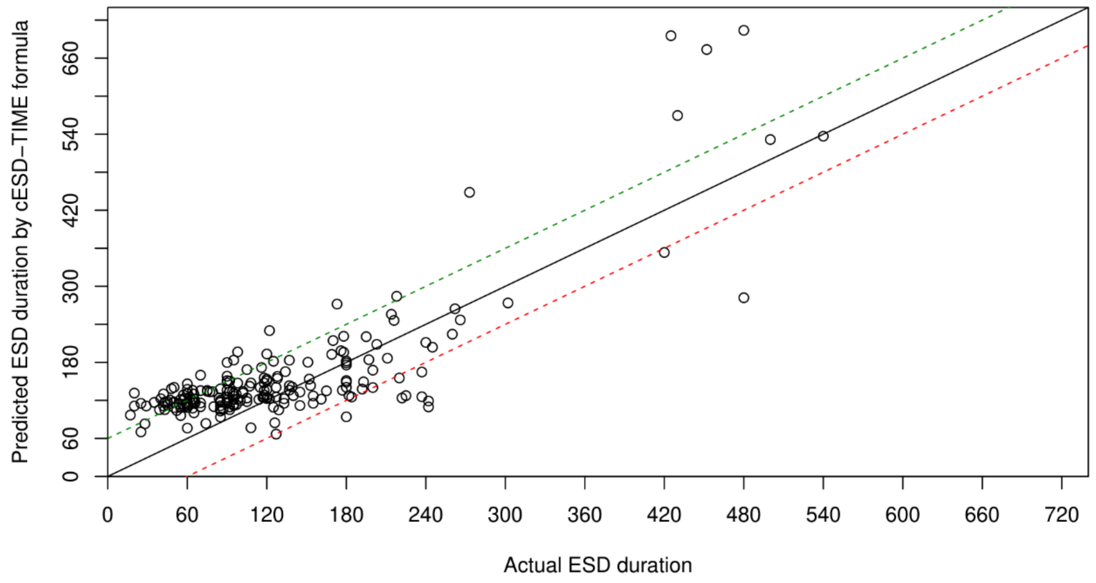

Supplementary Figure 5. Performance of the cESD-TIME formula in the Swedish validation cohort (n=199 procedures). *ESD:* endoscopic submucosal dissection

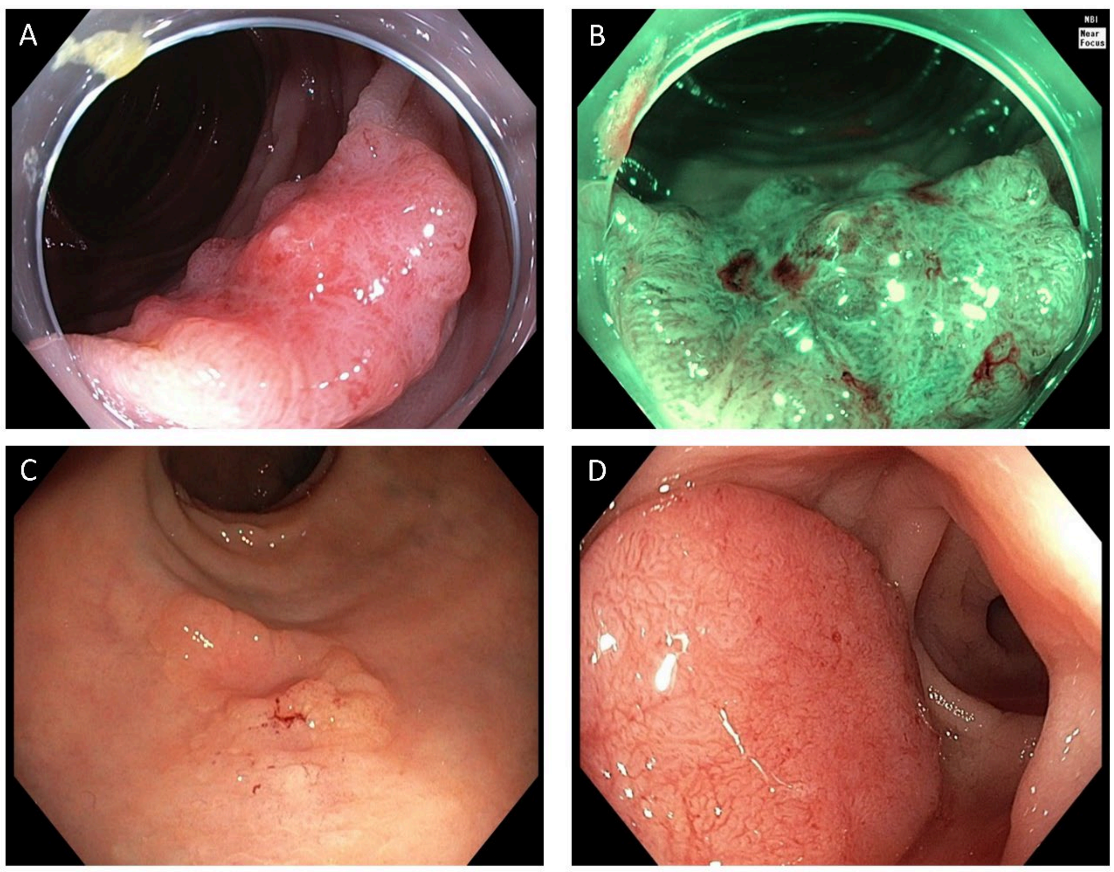

Supplementary Figure 6. Representative white-light (A) and advanced imaging (B) pictures of suspected CRC; (C) depression; (D) non-granular lateral spreading tumor with a luminal circumference of 40%.

Supplementary material

References

1. Charlson ME, Pompei P, Ales KL, MacKenzie CR. A new method of classifying prognostic comorbidity in longitudinal studies: development and validation. J Chronic Dis. 1987;40(5):373-83.

2. Bisschops R, East JE, Hassan C, Hazewinkel Y, Kaminski MF, Neumann H, et al. Advanced imaging for detection and differentiation of colorectal neoplasia: European Society of Gastrointestinal Endoscopy (ESGE) Guideline - Update 2019. Endoscopy. 2019;51(12):1155-79.

3. Hashiguchi Y, Muro K, Saito Y, Ito Y, Ajioka Y, Hamaguchi T, et al. Japanese Society for Cancer of the Colon and Rectum (JSCCR) guidelines 2019 for the treatment of colorectal cancer. Int J Clin Oncol. 2020;25(1):1-42.

4. Zwager LW, Bastiaansen BAJ, Montazeri NSM, Hompes R, Barresi V, Ichimasa K, et al. Deep Submucosal Invasion Is Not an Independent Risk Factor for Lymph Node Metastasis in T1 Colorectal Cancer: A Meta-Analysis. Gastroenterology. 2022;163(1):174-89.

5. Lugli A, Kirsch R, Ajioka Y, Bosman F, Cathomas G, Dawson H, et al. Recommendations for reporting tumor budding in colorectal cancer based on the International Tumor Budding Consensus Conference (ITBCC) 2016. Mod Pathol. 2017;30(9):1299-311.

6. Integraal Kankercentrum Nederland. Richtlijn Colorectaalcarcinoom (3.0) 2013 [updated 16-04-2014]. Available from: https://www.oncoline.nl/colorectaalcarcinoom.

7. Nederlandse Vereniging van Maag-Darm-Leverartsen. Nederlandse Richtlijn Coloscopie Surveillance 2013 [updated 28-05-2013]. Available from: https://www.mdl.nl/kwaliteitszaken/richtlijnen.

8. Yamashina T, Nemoto D, Hayashi Y, Fukuda H, Okada M, Takezawa T, et al. Prospective randomized trial comparing the pocket-creation method and conventional method of colorectal endoscopic submucosal dissection. Gastrointest Endosc. 2020;92(2):368-79.

9. Hayashi Y, Miura Y, Yamamoto H. Pocket-creation method for the safe, reliable, and efficient endoscopic submucosal dissection of colorectal lateral spreading tumors. Dig Endosc. 2015;27(4):534-5.

10. Arantes V, Albuquerque W, Freitas Dias CA, Demas Alvares Cabral MM, Yamamoto H. Standardized endoscopic submucosal tunnel dissection for management of early esophageal tumors (with video). Gastrointest Endosc. 2013;78(6):946-52.

11. Li B, Shi Q, Xu EP, Yao LQ, Cai SL, Qi ZP, et al. Prediction of technically difficult endoscopic submucosal dissection for large superficial colorectal tumors: a novel clinical score model. Gastrointest Endosc. 2021;94(1):133-44 e3.

Supplementary material

12. Rubin DB, Wiley I. Multiple imputation for nonresponse in surveys. Hoboken, N.J: Wiley-Interscience; 2004.

13. Riley RD, Ensor J, Snell KIE, Harrell FE, Jr., Martin GP, Reitsma JB, et al. Calculating the sample size required for developing a clinical prediction model. BMJ. 2020;368:m441.

14. Austin PC, Steyerberg EW. The number of subjects per variable required in linear regression analyses. J Clin Epidemiol. 2015;68(6):627-36.

15. Steyerberg EW. Clinical Prediction Models: A Practical Approach to Development, Validation, and Updating. Place of publication not identified: Springer Nature : Springer; 2019.

16. Steyerberg EW, Harrell FE, Jr. Prediction models need appropriate internal, internal-external, and external validation. J Clin Epidemiol. 2016;69:245-7.

17. Jacques J, Legros R, Wallenhorst T, Pioche M, French colorectal ESDg. Prediction of technically difficult, colorectal, endoscopic submucosal dissection: Is the procedural time a good endpoint? Gastrointest Endosc. 2021;93(6):1435-6.

18. Yahagi N, Maehata T. What is important for a smooth implementation of endoscopic submucosal dissection? Gastrointest Endosc. 2021;94(1):145-7.

19. Hayashi N, Tanaka S, Nishiyama S, Terasaki M, Nakadoi K, Oka S, et al. Predictors of incomplete resection and perforation associated with endoscopic submucosal dissection for colorectal tumors. Gastrointest Endosc. 2014;79(3):427-35.

Supplementary material

TRIPOD Checklist: Prediction Model Development and Validation

Section/Topic	Item		Checklist Item	Page
**Title and abstract**				
Title	1	D;V	Identify the study as developing and/or validating a multivariable prediction model, the target population, and the outcome to be predicted.	1
Abstract	2	D;V	Provide a summary of objectives, study design, setting, participants, sample size, predictors, outcome, statistical analysis, results, and conclusions.	1
**Introduction**				
Background and objectives	3a	D;V	Explain the medical context (including whether diagnostic or prognostic) and rationale for developing or validating the multivariable prediction model, including references to existing models.	2
	3b	D;V	Specify the objectives, including whether the study describes the development or validation of the model or both.	2
**Methods**				
Source of data	4a	D;V	Describe the study design or source of data (e.g., randomized trial, cohort, or registry data), separately for the development and validation data sets, if applicable.	2
	4b	D;V	Specify the key study dates, including start of accrual; end of accrual; and, if applicable, end of follow-up.	2
Participants	5a	D;V	Specify key elements of the study setting (e.g., primary care, secondary care, general population) including number and location of centres.	2
	5b	D;V	Describe eligibility criteria for participants.	2
	5c	D;V	Give details of treatments received, if relevant.	Suppl
Outcome	6a	D;V	Clearly define the outcome that is predicted by the prediction model, including how and when assessed.	2
	6b	D;V	Report any actions to blind assessment of the outcome to be predicted.	NA
Predictors	7a	D;V	Clearly define all predictors used in developing or validating the multivariable prediction model, including how and when they were measured.	2, 3
	7b	D;V	Report any actions to blind assessment of predictors for the outcome and other predictors.	NA
Sample size	8	D;V	Explain how the study size was arrived at.	Suppl
Missing data	9	D;V	Describe how missing data were handled (e.g., complete-case analysis, single imputation, multiple imputation) with details of any imputation method.	Suppl
Statistical analysis methods	10a	D	Describe how predictors were handled in the analyses.	Suppl
	10b	D	Specify type of model, all model-building procedures (including any predictor selection), and method for internal validation.	Suppl
	10c	V	For validation, describe how the predictions were calculated.	Suppl
	10d	D;V	Specify all measures used to assess model performance and, if relevant, to compare multiple models.	Suppl
	10e	V	Describe any model updating (e.g., recalibration) arising from the validation, if done.	Suppl
Risk groups	11	D;V	Provide details on how risk groups were created, if done.	NA
Development vs. validation	12	V	For validation, identify any differences from the development data in setting, eligibility criteria, outcome, and predictors.	2
**Results**				
Participants	13a	D;V	Describe the flow of participants through the study, including the number of participants with and without the outcome and, if applicable, a summary of the follow-up time. A diagram may be helpful.	Suppl. Fig.1
	13b	D;V	Describe the characteristics of the participants (basic demographics, clinical features, available predictors), including the number of participants with missing data for predictors and outcome.	3–5
	13c	V	For validation, show a comparison with the development data of the distribution of important variables (demographics, predictors and outcome).	Suppl tab 6
Model development	14a	D	Specify the number of participants and outcome events in each analysis.	5–6
	14b	D	If done, report the unadjusted association between each candidate predictor and outcome.	Suppl tab 4
Model specification	15a	D	Present the full prediction model to allow predictions for individuals (i.e., all regression coefficients, and model intercept or baseline survival at a given time point).	Table 4
	15b	D	Explain how to the use the prediction model.	5–7
Model performance	16	D;V	Report performance measures (with CIs) for the prediction model.	5–6
Model-updating	17	V	If done, report the results from any model updating (i.e., model specification, model performance).	7 Suppl
**Discussion**				
Limitations	18	D;V	Discuss any limitations of the study (such as nonrepresentative sample, few events per predictor, missing data).	7–8
Interpretation	19a	V	For validation, discuss the results with reference to performance in the development data, and any other validation data.	7–8
	19b	D;V	Give an overall interpretation of the results, considering objectives, limitations, results from similar studies, and other relevant evidence.	6–8
Implications	20	D;V	Discuss the potential clinical use of the model and implications for future research.	7
**Other information**				
Supplementary information	21	D;V	Provide information about the availability of supplementary resources, such as study protocol, Web calculator, and data sets.	Suppl Fig 4
Funding	22	D;V	Give the source of funding and the role of the funders for the present study.	8

*Items relevant only to the development of a prediction model are denoted by D, items relating solely to a validation of a prediction model are denoted by V, and items relating to both are denoted D;V. We recommend using the TRIPOD Checklist in conjunction with the TRIPOD Explanation and Elaboration document.

From: Collins GS, Reitsma JB, Altman DG, Moons KG. Transparent reporting of a multivariable prediction model for individual prognosis or diagnosis (TRIPOD): the TRIPOD statement. *BMJ.* 2015 Jan 7;350:g7594. doi: 10.1136/bmj.g7594.
